# Factors influencing the habitat choice of pangolins (*Manis* spp.) in low land of Nepal

**DOI:** 10.1002/ece3.8156

**Published:** 2021-09-23

**Authors:** Arati Shrestha, Santosh Bhattarai, Binod Shrestha, Narayan Prasad Koju

**Affiliations:** ^1^ Central Department of Environmental Science Tribhuvan University Kathmandu Nepal; ^2^ National Trust for Nature Conservation‐Biodiversity Conservation Center Chitwan Nepal; ^3^ Natural Resources Management Program Centre for Post Graduate Studies Nepal Engineering College Lalitpur Nepal; ^4^ Department of Psychology University of Washington Seattle WA USA

**Keywords:** burrow, habitat preferences, occurrence sites, PCA

## Abstract

Pangolins in the genus *Manis* are nocturnal, burrowing, insectivorous mammals listed as Critically Endangered or Endangered by the International Union for Conservation of Nature. Two species of pangolins are found in Nepal: the Chinese pangolin (*Manis pentadactyla*) and Indian pangolin (*Manis crassicaudata*). Despite having high conservation priority, little attention has been given to conservation interventions of both species of pangolins found in the Terai region (low land) of Nepal. The present study assesses habitat use and factors affecting the habitat choice of pangolins in low land (Terai), Nepal, focusing on Amritdharapani Community Forest of Chitwan district. Pangolin burrows were used as the indirect signs, and opportunistic sampling method was used to record the burrows. After the identification of all occurrence sites (burrows) in the field, random points were generated excluding the points where burrows were recorded for sampling of nonoccurrence sites. A total of thirty‐nine burrows were observed at elevations ranging from 301 to 413 masl. Burrows were frequently associated with northwest aspects, gentle slope (15°–20°), moderate canopy cover (51%–75%), red‐colored soil, and acidic soils with pH 6.5–7. The burrows were most common in areas with weak human disturbance (i.e., 1,500–1,700 m from settlements), 800–1,200 m from roads, and within 300 m from a water source and within 20 m from the nearest termitarium. Distance to settlement, distance to road, soil pH, and canopy cover were found to affect the habitat choice of pangolins in the study area.

## Graphical abstract

The present study assesses habitat use and factors affecting the habitat choice of pangolins in low land (Terai), Nepal. The research was focused on Amritdharapani Community Forest of Chitwan District. This study revealed distance to settlement, distance to road, soil pH, and canopy cover as major factors affecting the habitat choice of pangolins in the study area.

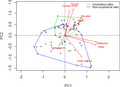

## 1 INTRODUCTION

Pangolins (family: Manidae) encompass eight extant species distributed discontinuously through tropical and subtropical Asia and Africa (Marler, 2016). Nepal lies in the transition zone between Palearctic and Oriental regions and hence is endowed with fauna characteristic of both regions, including the “Critically Endangered” Chinese pangolin (*Manis pentadactyla*) (Challender et al., 2019) and the “Endangered” Indian pangolin (*Manis crassicaudata*) (Baral & Shah, 2008; Jnawali et al., 2011; Kaspal, 2009; Mahmood, Akrim, et al., 2019; Mahmood, Challender et al., 2019). Both species have been upgraded to Appendix I of the Convention on International Trade in Endangered Species of Wild Fauna and Flora (CITES) due to the high threat of extinction (CITES, 2016), and both species have been accorded the highest degree of protection under the National Parks and Wildlife Conservation Act (NPWCA, 1973).

Pangolins have been reported throughout Nepal from Terai (lowland plain areas) to mid‐hill regions rising up to 3,000 m above sea level particularly in eastern Nepal and have occupied an array of natural and man‐made habitats including primary and secondary forests in both protected and nonprotected areas (Gurung, 1996; Sharma et al., 2020). They have been detected in open lands, riverine forests, sal (*Shorea robusta*) forest, mixed hardwood forests, bamboo forests, grasslands, and agricultural and degraded marginal lands near human settlements where there is availability of food, water, and sunlight (DNPWC & DoF, 2018; Jnawali et al., 2011; Suwal, 2011). A national pangolin survey carried out in 2016 revealed the distribution of pangolins in 43 districts of Nepal. The Chinese pangolin and Indian pangolin were distributed in 25 and 7 districts, respectively (DNPWC & DoF, 2018). Chinese pangolins were more widely distributed, occurring up to 2000 m in Nepal's central and eastern regions. Indian pangolins occurred only in a small part of southern Nepal, the eastern foothills, and the Terai region, mostly in tropical and subtropical forests and mostly below 500 m, although recently recorded at an elevation of 675 masl (Baral & Shah, 2008; DNPWC & DoF, 2018; Jnawali et al., 2011; Suwal et al., 2020). Baral and Shah (2008) has discussed the distribution of Chinese pangolins with upper altitudinal gradients of 2,500 m. The occurrence of pangolin is greatly influenced by various habitat covariates such as canopy coverage, soil type, aspect, and ground vegetation coverage, distance to water source, human settlements, agricultural land, and anthropogenic factors such as poaching as well (Bhandari & Chalise, 2014; Gurung, 1996; Katuwal et al., 2017; Sharma et al., 2020; Suwal et al., 2020).

Pangolin habitat use, and characteristics of used or associated habitats appear to differ according to environmental conditions (Karawita et al., 2018), and identification of suitable habitat and potential distribution is essential for developing conservation strategies for these species (Katuwal et al., 2017). The current study assessed pangolin habitat preferences to aid in preventing the loss of prime pangolin habitats in Amritdharapani Community Forest of Chitwan District, Nepal.

## 2 METHODOLOGY

### 2.1 Study area

**FIGURE 1:**
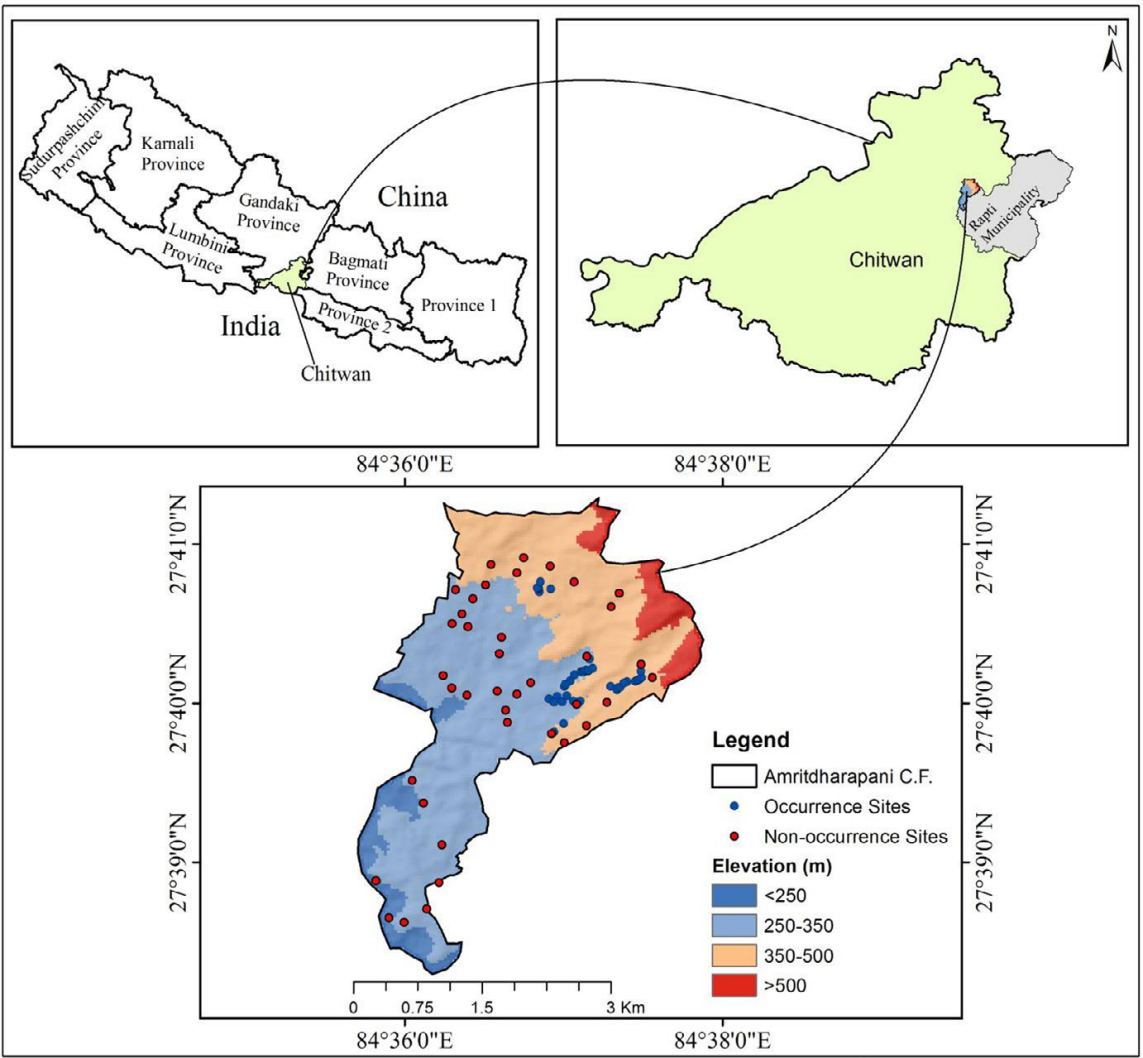
Map showing the location of the study area

The Chitwan District is located at low land (Terai) in the Bagmati Province of Nepal at 83°55′45″ to 84°48′15″ East longitudes and 27°52′6″ to 27°46′ North latitude, an area of tropical and subtropical monsoon climate with high humidity (Poudel, 2014). Chitwan is famous for Chitwan National Park, a protected area listed as a UNESCO world heritage site. Evidence of the occurrence of both the Chinese pangolin and the Indian pangolin was recorded from the Chitwan District (DNPWC & DoF, 2018; Kaspal, 2009; Suwal et al., 2020). The study was carried out in Amritdharapani Community Forest located in Rapti Municipality of Chitwan District (Figure 1). It extends from 27°38′20″N to 27°41′43″N latitude and 84°35′47″E to 84°37′52″E longitude and covers an area of 853.182 hectares. This forest was comprised of the typical character of the Terai region of Nepal: mixed vegetation of different tree species dominated by *Shorea robusta*, *Lagerstroemia parvifolia*, *Careya arborea*, *Syzygium operculatum*, *Rhus wallichii*, *Eugenia jambolana,* etc. Many wildlife species such as *Panthera pardus, Manis pentadactyla, Axis, axis, Herpestus edwardsi, Sciuridae* sp., *Macaca mulata*, etc., are also found in this community forest.

### 2.2 Methods

The distribution of pangolins was determined through field surveys between February and April 2019. Each survey was conducted in two phases. Initially, the whole community forest was thoroughly searched by walking along different available trails by a team of at least four people to explore the pangolin signs. Two wildlife technicians and forest guide having knowledge about pangolin signs and potential areas of burrow distribution were also in the team for exploration and confirmation. Opportunistic sampling was then used to collect information related to pangolin burrows. The pangolins are highly elusive and nocturnal animals, notably difficult to observe in the wild (Kaspal, 2009). Therefore, its presence in the study area was inferred based on indirect signs such as burrows, footprints, tracks, and fecal matter (Akrim et al., 2017; Mahmood et al., 2017; Karawita et al., 2018; Waseem et al., 2020). Burrows were classified as “old” or “new” following Suwal (2011). Burrows with compact and dry soil with dry leaves and lacking signs such as fresh scratches, footprints, and fecal pellets were classified as old burrows, while burrows with loose soil without dry leaves around it, having signs of fresh scratches, footprints, and fecal pellets, are classified as new burrows. The occurrence sites, that is, sites having burrows, were identified in the field by an intensive survey of the study area. Once these sites were identified, 39 random points were generated in ArcGIS 10.3 (excluding the points where burrows were recorded in the initial survey) for sampling of nonoccurrence sites (sample points without burrows). All of them were visited for sampling where no sign of burrow was recorded and listed as nonoccurrence sites.

In each of the occurrence and nonoccurrence sites selected for sampling, habitat covariates were recorded including elevation, slope, aspect, soil pH, soil type based on color, canopy cover (%) above the burrow, distance to the nearest water source, and distance to nearest termitarium. Besides, distance to the nearest settlement and distance to the nearest road were also recorded, to index anthropogenic effects on the occurrence of pangolins (DNPWC & DoF, 2018; Katuwal et al., 2017; Sharma et al., 2020). In each occurrence site, the geographical coordinates of the burrow location were recorded using a GPS receiver (Garmin eTrex 10). Vegetative canopy cover was determined using a spherical densiometer (Lemmon, 1956). The soil sample was collected, and soil pH was determined later in the laboratory by using a pH meter. The slope at the center of each site was measured using a clinometer. Burrow location and random point generated within GIS were considered as the site center for the occurrence and nonoccurrence site, respectively. Covariates such as distance to the nearest settlement, road, and water body were based on GPS coordinates retrieved using ArcGIS 10.3.

### 2.3 Statistical analysis

R software version 3.5.1 was used for all statistical analyses. As the data were sufficiently normally distributed and the sample size was greater than 30, z tests were carried out to test the hypothesis that pangolin occurrence sites differed in habitat make‐up from nonoccurrence sites by comparing the difference in mean for each habitat variable. Principal component analysis (PCA) was used to quantitatively and qualitatively describe pangolin occurrence and nonoccurrence sites concerning the variance in measured habitat variables, and to understand how these sites diverge from each other (Shenoy et al., 2006). The PCA was based on a correlation matrix, which can be preferable to a covariance matrix when the focal variables have different units (Jackson, 1991). The variables that were entered into PCA were elevation (m), slope (°), soil pH, canopy coverage (%), distance to the water source (m), distance to nearest termitarium (m), distance to settlement (m), and distance to the road (m).

## 3 RESULTS

A total of 39 burrows were recorded from Amritdharapani Community Forest, out of which 35.89% (*n* = 14) were old burrows, while 64.10% (*n* = 25) were new burrows.

Pangolin burrows were distributed within an elevation range of 300–413 masl, and 30.77% of total burrows occurred within 320–340 masl. Burrows was not recorded below an elevation of 300 masl, and the distribution of the burrow was related significantly with elevation (*p* = .0015). Pangolin burrows were distributed on slopes of 5°–22°, and 56.41% of total burrows occurred on slopes of 15°–20°. No burrows were recorded on slopes less than 5° or greater than 25°, and burrow occurrence was related significantly to slope (*p* = .0001). 56.41% of burrows were recorded on slopes with northwest aspect, and burrows were not recorded on slopes facing east. 74.36% burrows were recorded in areas having canopy cover between 51% and 75%, while no burrows were recorded in areas where canopy cover was between 0% and 50%. Similarly, 56.41% burrows were recorded in red‐colored soil, followed by brown‐colored soil (33.33%), and 2.56% of burrows were recorded in reddish‐brown, blackish‐brown, and yellow soil. 58.97% of burrows were recorded in neutral soil (pH 6.5–7), 30.77% in slightly acidic soil (pH 6–6.5), and only 5.13% in alkaline soil (pH > 7). Canopy cover and soil pH showed a significant relation to burrow distribution (*p <* .05).

Furthermore, the burrows were recorded within 40–800 m from the nearest water source, with 25.64% of burrows distributed within 200–300 m from the water source, and only 2.56% at a distance of 700–800 m from the water source. No burrows were recorded beyond 800 m from a water source. The number of burrows decreased with increasing distance from the nearest water source (*p* = .003). Pangolin burrows were recorded within a range of 700–1900 m from the nearest human settlement, and 30.77% of them were recorded at 1,500–1,700 m from a settlement. Only 7.69% of the burrows were recorded at 700–900 m from the nearest human settlement, and there was no significant relationship (*p* > .05) between distance to settlement and burrow distribution.

Similarly, burrows were observed between 600 and 1,800 m from the nearest road, and 23.08% recorded at 800 to 1,200 m from the nearest road. Only 10.26% of burrows were observed relatively close to (600–800 m) or far from (1,600–1,800 m) the nearest road, and there was no significant relationship (*p* > .05) between distance to the nearest road and burrow distribution.

Burrows were found within 0–140 m of the nearest termitarium (food source). 43.6% burrows were observed within 20 m of the nearest termitarium, and only 2.6% were recorded at distances greater than 120 m from the nearest termitarium. There was a significant relationship between distance to nearest termitarium and burrow distribution (*p* = .0007).

**FIGURE 2:**
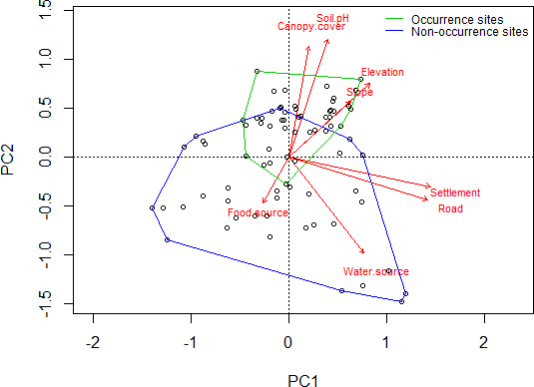
PCA plot showing factors affecting the distribution of burrows

Using PCA, the first four principal components together explained 86.72% of the variation in the data (Table 1). PC5 (7.49%) and the other three principal components were excluded from the model for the sake of simplicity. The first principal component explained 33.42% of the variance (Table 1) and was positively influenced by distance to road and distance to settlement, while the second principal component explained 28.47% of the variance and was positively influenced by soil pH and canopy cover (Table 1). Most of the occurrence sites clustered in the first and second quadrants, whereas the nonoccurrence sites clustered in the third and fourth quadrants. Distance to the road, distance to settlement, soil pH, and canopy cover had large impacts on the distribution of data (Figure 2). Distance to food source was negatively correlated with canopy cover, soil pH, elevation, and slope.

**TABLE 1:** Character loading, eigenvalue, and percent variance explained by the first four principal components

Variables	PC1	PC2	PC3	PC4
Elevation	0.97	0.88	−0.93	0.33
Slope	0.74	0.67	−0.01	−1.42
Soil pH	0.46	**1.42**	0.41	0.12
Canopy cover	0.24	**1.34**	0.12	0.59
Water source distance	0.89	−1.16	0.69	0.19
Settlement distance	**1.69**	−0.35	−0.13	0.09
Road distance	**1.66**	−0.51	−0.09	0.11
Nearest termitarium distance	−0.31	−0.55	−1.44	−0.0.04
Eigenvalues	2.67	2.28	1.17	0.82
Total variances explained	33.42%	28.47%	14.63%	10.20%

Note

The bold number represents for loading factors of a variable over 1.

## 4 DISCUSSION

Suwal et al. (2020) predicted the elevation range of the pangolin habitat to lie between 132 and 2,704 masl and classified the habitat below 500 masl and above 1,750 masl as less suitable for the taxon. Our study revealed that burrows were most common from 300 to 350 masl in the study area and distributions of burrows are significant to elevation. Gathorne‐Hardy et al. (2001) explained that the abundance of termite mounds decreases with increased elevation. Burrows were sparsely distributed above 400 masl. Bhandari and Chalise (2014) and Katuwal et al. (2013) suggested pangolins might prefer tropical and subtropical climatic zones. Lamichhane and Pokhrel (2019) recorded maximum burrows at 500–600 masl and found a decrease in burrow numbers with an increase in elevation above 600 masl. The study carried out by Dorji et al. (2020) also supported a negative relationship between elevation and number of burrows.

Pangolins mostly prefer slopes less than 50° (Wu et al., 2003), and in this study, most burrows were recorded on slopes between 15° and 20° showing significant relation to slope. Our result is similar to the findings of Karawita et al. (2018) and Sharma et al. (2020), which showed the preference of less than 30° slope and 15°–22° slope, respectively, by the taxon. The preference of slope less than 25° by the species might be for the easy movement in the area and for avoidance of terrain slope (Acharya et al., 2021). Slopes in this range may facilitate digging (Dorji, 2016) and reduce rain‐mediated soil erosion. Maurice et al. (2019) also recorded more burrows in gentle and steep slopes but very few in very steep slopes. But this result contradicts the findings of studies by Wu et al. (2004) and Suwal et al. (2020), which report pangolin preference for slopes of 30–60° and 30–50°, respectively, for making burrows. Burrows were not recorded in flat areas. Our results also showed the strong correspondence between elevation and slope as depicted in the PCA plot. In the study area, it was observed that with the increase in elevation, the slope also increased. Thus, it supported the findings of less burrows with increase in both elevation and slope.

Although aspect was not a statistically significant predictor of burrow location in our study, most of the burrows encountered were on slopes with northwest and southwest aspects, in general agreement with several other studies. Bhandari and Chalise (2014) reported similar patterns in Nagarjun Forest of Shivapuri National Park. Dhakal (2016) also reported the pangolin's preference to north‐facing slopes. The pangolin prefers the west slope probably for getting sunlight before foraging (Acharya et al., 2021). However, Suwal (2011) reported the random distribution of burrows in different aspects. Thapa et al. (2014) also observed preference of southwest aspect by pangolins for digging burrows. Gurung (1996) recorded pangolin preference for south‐facing slopes. A study by Dhital et al. (2020) in the Nagarjun forest of Shivapuri National Park showed pangolin preference for eastern aspects. The openings of pangolin burrows often face the sun (eastward) to make digging easier and to maintain burrow temperature in winter (Wu et al., 2004). The preference for certain aspects in certain locations might be influenced by local climatic conditions, available food, water, and degree of human interference (Dhakal, 2016).

Burrows were mostly found in areas having canopy cover between 51% and 75%, which is similar to another study by Rai et al. (2019) and Suwal et al. (2020) in Nepal, who reported that pangolins prefer habitats with medium canopy coverage rather than forests that are too dense or too sparse. The distribution of burrows was found to be significant with canopy cover. Other studies carried out in districts located in the mid‐hills of Nepal (Bhandari & Chalise, 2014; Dhakal, 2016; Dhital et al., 2020) have shown pangolin preference for burrowing under low canopy cover (25%–50%), which contradicted this study. Thapa et al. (2014) in a study of the Taplejung District recorded more burrows associated with a canopy cover of 0%–25% and the lowest number of burrows under canopy cover of 50%–75%. The variation observed may be due to the location of the study areas in different geographical locations. Bhandari and Chalise (2014) also found that the lowest burrow numbers occurred in areas having more than 75% canopy cover, which corroborated our study. Wu et al. (2003) in a winter study of Dawuling Natural Reserve recorded that pangolins avoid vegetation density over 75% and fewer than 30%. Karawita et al. (2018) found burrows were more frequent in areas with moderate canopy cover (41%–70%). In places where pangolins prefer low canopy cover sites, they might be influenced by prey availability because pangolin's prey; that is, termites occur more in dry areas than in wet areas. The dense forest canopy helps in the protection of forest moisture, and it also determines the density of the understory (Maurice et al., 2019).

Most of the burrows were observed in red‐colored soil, which corresponded to findings of the other studies (Acharya, 2001; Sharma et al., 2020) Sharma et al. (2020) suggested more occurrences of pangolin burrows in red soil as a result of increased food availability compared with brown soils. But the observation contradicts the findings of Kaspal (2009), Suwal (2011), and Suwal et al. (2020), which showed the preference for brown‐colored soil for digging burrows. Kaspal (2009) found the burrows with more depth and diameter in red‐colored soil than in brown‐colored soil.

The majority of burrows (56.41%) were found in soils having acidic or neutral pH. The distribution of burrows showed a significant relationship with soil pH. Rai et al. (2019) also recorded more burrows in soil having acidic and more or less neutral pH value. The presence of more burrows in acidic soil might be supported by the fact that its prey termites are mostly found in acidic and weakly alkaline soils with pH values between 3.5 and 8.7 and increase in soil pH might lead to termite inactivation (Li et al., 2017). This is supported by the dominance of red‐colored soil in sites where burrows were observed. Red‐colored soil is comparatively more acidic than brown‐colored soil. Soil pH helps to determine what type of vegetation will be available in the area, and indeed, canopy cover corresponded to soil pH. Termite abundance tends to decrease in abundance in acidic soil conditions.

The gradual decreases in the number of burrows were observed with the increase in distance to water source showing significant relation between burrow distribution and distance to a water source, which is similar to the findings of Dorji et al. (2020). From this, it can be presumed that the distance to the water source acts as one of the factors affecting the distribution of pangolins. Katuwal et al. (2013) recorded a higher number of burrows in an area within a distance of 100 m from the water source and observed only one burrow at 700‐m distance from the water source. Katuwal et al. (2017) recorded more pangolin occurrence in area within 200 m distance from water source. Karawita et al. (2018) found burrows were more frequent in areas with distance 100–200 m from the water source. The presence of burrows at the proximity of the water source might be for avoiding predators and conserving vital energy while walking a long distance for water (Bista et al., 2017). As the pangolins need water for themselves and ants, termites, and other insects also prefer moist habitats, this might be another reason behind the presence of burrows near a water source (Katuwal et al., 2013).

The number of burrows increased with the increase in distance from settlement area and road. This finding has been supported by other studies carried out in Nepal. Katuwal et al. (2017) recorded more pangolin occurrence in the area with less disturbance (typically >1,000 m from human settlements and road). In contrast to this, Sharma et al. (2020) recorded 51% of occurrence plots within a 1000‐m distance from settlement areas. Similarly, a study by Karawita et al. (2018) revealed burrow distribution was greater in an area with greater human disturbance (<200 m) and has decreased with increased distance from human settlement. Katuwal et al. (2013) recorded more burrows close to the settlement area with a distance of at least 50–200 m. Wu et al. (2003) observed pangolins dug burrows away from human habitation, which suggests an inclination to avoid disturbances. The study carried out by Gurung (1996) shows that human encroachment in the preferred habitats of the pangolin is the reason behind the decline of species in Nepal. Areas close to settlements experience more frequent livestock and human activity, and pangolins have been observed to leave their burrows following such activities (Katuwal et al., 2017). The collection of fallen logs by humans and excessive trampling by larger‐hoofed cattle result in detrimental effects on the survival of pangolins due to disturbance and decrease in prey availability. Apart from that, foot trails and settlements can ease the poaching of the species (Katuwal et al., 2017). Katuwal et al. (2013) observed burrows close to the small walking trails of the humans with more recorded within the distance of 0–50 m.

The gradual decreases in the number of burrows were observed with the increase in distance from the nearest termitarium showing significant relation between burrow distribution and distance to the nearest termitarium. This indicates the distance to the nearest food source has a significant impact on the distribution of burrows. The result can be supported by the fact that pangolins are specialist species with food specialization, that is, only eating ants and termites, and for the pangolins to thrive, there should be a natural abundance of its prey species, and hence, their habitat location is also linked with their prey species (Maurice et al., 2019; Wu et al., 2004). The burrows are also dug near the food source for the easy availability of prey during the winter months (Heath & Vanderlip, 1988). The high termite species abundance and richness favor pangolin occurrence (Swart et al., 1999). In the study area, dead fallen logs and leaf litter were observed during field visits, which provide favorable habitats for ants and termites.

The studies on habitat preferences of the pangolin within the country and across its range countries have also reported variable findings (Bhandari & Chalise, 2014; Katuwal et al., 2017; Lamichhane & Pokhrel, 2019; Sharma et al., 2020; Suwal et al., 2020), which is supported by our result too. The study area is located in the low land of Nepal not having a slope greater than 40° and elevation range below 600 masl, and the difference in climate and topography might have resulted in the variation in the habitat preference by the species compared with the other studies that were carried in mid‐hill region of Nepal.

Our results supported the hypothesis that occurrence sites were significantly different from nonoccurrence sites in several of the variables we measured. Out of the eight variables used, distance to settlement, distance to road, soil pH, and canopy coverage were the most significant factors responsible for burrow distribution in the study area. However, Katuwal et al. (2013) observed no marked effect of distance to settlement and road in the distribution of burrows. Suwal et al. (2020) reported elevation as the major influential variable in determining suitable habitat for pangolins. Aryal and Poudel (2018) reported that the elevation, canopy coverage, and presence of *Schima wallichii* as dominant species are the major habitat variables affecting the distribution of the burrows in their study at Nagarjun and Ranibari forests, Kathmandu. The main factors determining the presence and habitat choice of pangolins are the availability of prey and water, but it is found to vary depending on the species (Maurice et al., 2019). Similarly, some degree of uncertainty was observed on the major and most favorable ecosystem preferred by pangolins (Kingdon et al., 2013). Detections of pangolins close to the settlement area suggest that anthropogenic activities may play a major role in their survival (Katuwal et al., 2017). Proximity to human settlements has proved to be detrimental for pangolins as hunters often capture pangolins near their burrows (Zhang et al., 2017). According to Katuwal et al. (2017), canopy coverage and water sources played a positive role in determining the occurrence of pangolin. In the closed‐canopy forest, prey species are highly abundant and there is less chance of erosion (Katuwal et al., 2017). According to Mahmood et al. (2014) and Pabasara et al. (2015), habitat features such as tree species composition, vegetation cover, presence of water source, and soil characteristics are the parameter that helps to characterize the habitat of pangolins. Various associated environmental features such as canopy coverage and elevation are likely to be site‐specific or habitat‐specific for pangolins (Karawita et al., 2018).

## 5 CONCLUSION

The study highlights the habitat use and major factors that affect the habitat choice of pangolins in Amritdharapani Community Forest of the Chitwan District. The study showed the preference of lower elevations, gentle slope, northwest aspect, moderate canopy coverage, reddish soil, acidic soils, area with less human disturbance, and area having easy access to water and food by the pangolins for digging burrows. Distance to settlement, distance to road, soil pH, and canopy cover were found to affect the habitat choice of pangolins in the study area. This study can act as a baseline, which can assist in the conservation planning of the pangolin species at low lands of Nepal.

## CONFLICT OF INTEREST

None declared.

## AUTHOR CONTRIBUTIONS

**Arati Shrestha:** Conceptualization (lead); data curation (lead); funding acquisition (lead); investigation (lead); methodology (lead); project administration (lead); writing–original draft (lead); writing–review and editing (lead). **Santosh Bhattarai:** Conceptualization (equal); data curation (equal); funding acquisition (equal); methodology (equal); project administration (equal); supervision (supporting); writing–original draft (supporting); writing–review and editing (supporting). **Binod Shrestha:** Conceptualization (supporting); data curation (supporting); funding acquisition (supporting); methodology (supporting); project administration (supporting); writing–original draft (supporting); writing–review and editing (supporting). **Narayan Prasad Koju:** Conceptualization (equal); data curation (equal); formal analysis (equal); funding acquisition (supporting); investigation (equal); methodology (equal); project administration (equal); supervision (lead); writing–original draft (supporting); writing–review and editing (equal).

## Data Availability

The data associated with this manuscript are available at: https://datadryad.org/stash/share/zD‐q7O_8acIe035PggIZzY5PKVwcazC1qi4gDYU7hUU
